# How to do qualitative research? Qualitative research methods

**DOI:** 10.1111/1471-0528.17150

**Published:** 2022-04-17

**Authors:** Elaine Denny, Annalise Weckesser

**Affiliations:** ^1^ Faculty of Health, Education and Life Sciences Birmingham City University Birmingham UK

**Learning points**

- The main methods used in qualitative research are interview, focus group and observation.
- Recruitment is purposive, or strategic, in that the aim is to achieve a sample that is relevant to the research question.
- Data are collected until saturation of themes and insights is reached.

Qualitative research begins with one or more relatively broad research questions that may be revised iteratively as the research is carried out to narrow the research aim or purpose. This is different from quantitative research, where a narrow research question is set at the start and remains fixed. For example, the aim of a study may be to explore the experiences of women who are pregnant while living with epilepsy. The initial research question may be ‘How do women with epilepsy experience pregnancy?’ However, from preliminary findings this may change to ‘How do women manage their epilepsy during pregnancy?’

There are three main methods used in qualitative research.

The first and most commonly used is interviewing. Semi‐structured interviews contain pre‐set, open‐ended questions, with further questions emerging from the discussion. Unstructured interviews cover a few issues in great depth, for example they can be used for life history narratives.

Focus groups are group discussions facilitated by a researcher, who will have guidelines to focus the group. Data collection consists of group interaction as well as discussion content. They can be stand alone, but more commonly are used to clarify or extend data collected by other methods.

Both interviews and focus groups tend to be flexible and non‐standardised, with greater interest in the participants' perspectives and experience than for quantitative research. However, it is important that flexibility does not result in asking leading questions.

Another method is observation, which is the act of watching social phenomena in real‐world settings, recording what people do, rather than what they profess to do. The observer may be part of the scene being observed (participant observation) or stand outside it (non‐participant).

Sampling for qualitative research tends to be purposive (that is recruitment on the basis of a shared experience that is relevant to the research question), convenience (based for example on accessibility or cost) or snowballing (where a few individuals from the target population will connect the researcher with their network). In the example above, women were purposefully recruited as pregnant, living with epilepsy and willing to be interviewed about how this impacted upon their lives (Weckesser & Denny. *Soc Sci Med* 2017:185;210–17).

The amount of data collected in qualitative research is not fixed or calculable, but continues until saturation is reached. That is, data are collected until emerging concepts have been explored and additional data are not producing fresh insights (Bryman. *Social Research Methods*, 3rd edn. Oxford: Oxford University Press; 2008). Generally speaking, the study sample size tends to be much smaller when compared with quantitative research.

Interviews and focus group discussions are usually audio‐recorded, with the consent of participants, and then transcribed verbatim. Written notes may also be made by the researcher for use in analysis. For the method of observation, extensive field notes are recorded during and after the event. Copious data are usually generated, which need to be organised for analysis, which is the focus of the next article in this series.

## CONFLICT OF INTEREST

None declared. Completed disclosure of interests form available to view online as supporting information.

## AUTHOR CONTRIBUTION

Elaine Denny and Annalise Weckesser contributed equally to the paper.

## USEFUL RESOURCES

Britten N. Qualitative research: qualitative interviews in medical research. *BMJ* 1995;311:251–3.

Kitzinger J. Qualitative research: introducing focus groups. *BMJ* 1995;311:229.

Savage J. Participative observation: standing in the shoes of others? *Qual Health Res* 2000;3:324–39.

## Supporting information


**Appendix** S1

**Appendix** S2

## Data Availability

Not applicable

